# Changes in the Physicochemical Properties, Antioxidant Activity and Metabolite Analysis of Black Elephant Garlic (*Allium ampeloprasum* L.) during Aging Period

**DOI:** 10.3390/foods12010043

**Published:** 2022-12-22

**Authors:** Se-Hyun Nam, Young-Sil Han, Ki-Hyeon Sim, Seung-Ok Yang, Myung-Hyun Kim

**Affiliations:** 1 Department of Food & Nutrition, Sookmyung Women’s University, Cheongpa-ro 47-gil 100, Yongsan-gu, Seoul 04310, Republic of Korea; 2 Traditional Culinary Culture, Graduate School of Arts, Sookmyung Women’s University, Cheongpa-ro 47-gil 100, Yongsan-gu, Seoul 04310, Republic of Korea; 3 National Instrumentation Center for Environmental Management, Seoul National University, 1 Gwanak-ro, Gwanak-gu, Seoul 08826, Republic of Korea

**Keywords:** elephant garlic, black garlic, antioxidant, metabolite, aging period

## Abstract

This study investigated the effects of the aging period on the black elephant garlic (*Allium ampeloprasum* L.) manufacturing process. Black elephant garlic is a processed elephant garlic product prepared by high-temperature and high-humidity treatment for 40 days. The proximate composition (moisture, crude lipid, crude protein, carbohydrate, and ash), minerals, color values, reducing sugars, pH, total polyphenol contents, total flavonoid contents and antioxidant activities of elephant garlic and black elephant garlic were evaluated. The browning intensity of elephant garlic increased with the aging period, but the browning reaction terminated after aging for 30 days, exhibiting the same browning level. Reducing sugars increased over the aging period until 20 days, and then decreased with the aging period, in contrast to the pH, which decreased from 6.47 to 3.68 over the aging period. Antioxidant components, including the total polyphenol and total flavonoid contents of black elephant garlic, increased significantly until day 30 of aging. From the metabolite profiles determined through GC/MS analysis, it was confirmed that primary metabolites related to antioxidant components, such as lactic acid and 5-hydroxymethyl-2-furoic acid, were generated during the aging process of elephant garlic.

## 1. Introduction

Allium vegetables have been used as folk medicine since ancient times [1]. The Allium genus has over 600 members which differ in maturing, color and taste [2]. However, they are similar in biochemical content [1]. Garlic (*Allium sativum* L.) is an important spice with a distinctive odor that has been widely consumed worldwide for thousands of years, and is particularly popular in Europe, Asia, and Africa [3]. Some of its many bioactive components are organic sulfides, saponins, phenolic compounds, and polysaccharides. Elephant garlic (EG) (*Allium ampeloprasum* L.) resembles leek (*A. ampeloprasum* var. *porrum* (L.) J. Gay) and common garlic in terms of shape and flavor, although it is three times larger than standard garlic [3,4]. Also called giant garlic, Big Tex garlic, Tahiti garlic, or great-headed garlic, EG can weigh up to 450 g, has a milder flavor than garlic and has a sweet taste; thus, it is used as a substitute for garlic in many countries [1]. The most important features of EG are its much lower contents of fiber and alliin, the sulfur-containing compound responsible for the strong taste of garlic, conferring this plant with greater digestibility and a much more delicate taste than standard garlic [5]. Black garlic, an aged garlic preparation, has recently been introduced to the Asian market as a health product [6]. Black garlic is formed by aging whole garlic at a high temperature and high humidity, causing the garlic to turn black because of browning compounds [7]. Furthermore, black garlic does not exude a strong off-flavor, in contrast to fresh garlic. During the heating process, various physicochemical changes occur, such as changes in color, texture and flavor due to nonenzymatic browning reactions, such as the Maillard reaction and caramelization, which, in turn, alter the nutrient content [8]. These reactions are interconnected and produce the black color, sweet taste, sour taste and jelly-like texture of BG [9,10]. Studies have proven the bioactivities of black garlic, such as antioxidation and antiobesity, anti-inflammation, cancer prevention, hepatoprotection, hypolipidemia, antiallergy, immunomodulation, cardiovascular prevention, and neurodegenerative protection [11,12,13,14].

When elephant garlic is processed into black elephant garlic (BEG), TPC and TFC are increased, and S-allyl-L-cysteine, S-methyl-L-cysteine, diallyl sulfide, diallyl-disulfide, etc. are produced, and thus it has higher physiological activity than EG [15]. Recently, studies on antioxidant activity and antidiabetic activity have been conducted with interest in EG, but a comprehensive assessment of the health-beneficial effects of BEG is currently deficient [16]. In addition, there is limited information about the quality indicator content changes and the quality formation characteristics in EG during the aging process. It is considered important to measure physiochemical properties, antioxidant activity and metabolite analysis, which show quality formation characteristics in the aging process of EG. These results can contribute to understanding the role of aging periods in the quality formation of EG. When EG is aged, the possibility of using it as a new functional food material can be confirmed by measuring the general components, color, pH, etc., which are newly created or increased, and comparing and analyzing them according to the aging period.

In this study, BEG was produced from fresh EG by fermentation at 70 °C and 90% relative humidity for up to 40 days. Considering the various physicochemical changes that occur during the aging process, we hypothesized that there should be optimum aging conditions to maximize the antioxidant properties of BEG. In this regard, the objective of the study was to identify the physicochemical properties of BEG during 40 days of aging and determine the optimum aging period for maximum antioxidant properties, and also to propose the possible applications as a functional food product to future prospects.

## 2. Material and Methods

### 2.1. Samples

EG was purchased from Gimpo Agricultural Association, Republic of Korea, in June 2020. EG was prepared based on the method described in [17]. EG was aged in a hygrostat chamber (KCL2000W, Tokyo Rikakikai Co., Ltd., Tokyo, Japan) at 70 °C in 90% relative humidity for 10, 20, 30 and 40 days. EGs according to the aging period were freeze-dried (MCFD 8508, Ilshin Bio Base, Yangju, Republic of Korea) for standardization and pulverized with a grinder (EV-GB 8000, Everhome, Seoul, Republic of Korea) and used in the experiment.

### 2.2. Materials

3,5-dinitrosalicylic acid (DNS), Folin–Ciocalteu’s phenol reagent, 2,2-diphenyl-1-picrylhydrazyl (DPPH), methoxyamine hydrochloride, *N*, *O*-bis trimethylsilyl trifluoroacetamide (BSTFA), pyridine (anhydrous 99.8%), methanesulfonic acid glucose, gallic acid, rutin, Trolox and ascorbic acid were purchased from Sigma-Aldrich (St. Louis, MO, USA). Fluoranthene was purchased from Supelco (Bellefonte, PA, USA). All chemicals and reagents used in this study were of analytical grade.

### 2.3. Sample Preparation and Metabolite Analysis by GC/MS

The metabolite analysis was prepared according to Shin et al. [18]. Approximately 1 g of freeze-dried powder samples according to the aging period was mixed with 30 mL of 70% MeOH using a sonicator (Powersonic610, Hwashin Technology, Seoul, Republic of Korea) for 30 min. After filtration with 0.2 μm microporous membranes, 150 μL of the collected supernatant was transferred into a gas chromatography vial and dried using a centrifugal vacuum concentrator (Hypervac vc2200, Bmskorea, Gimpo, Republic of Korea). For the gas chromatography mass spectrometry (GC/MS) analysis of EG compounds according to the aging period, dried extract samples were redissolved and derivatized by the addition of 50 μL of 20 mg/mL methoxyamine hydrochloride in pyridine and incubated at 30 °C for 90 min for oxidation. Subsequently, 50 μL BSTFA was added to each sample for trimethylsilylation derivatization, as well as 30 μL of fluoranthene (1000 μg/mL in pyridine as an internal standard), and the mixture was heated at 60 °C for 30 min. Additionally, it was then subjected to GC/MS analysis. Each 1.0 μL aliquot of derivatized sample was injected in a 30:1 split ratio into a Trace 1310/ISQ LT GC/MS system (Thermo Scientific, Waltham, MA, USA) equipped with DB-5MS capillary column (60 mm × 0.25 mm × 0.25 μm, Agilent J & Scientific, Santa Clara, CA, USA). Helium was used as the carrier gas with a constant flow rate of 1.5 mL/min. The temperature program was as follows: the initial temperature was 50 °C, held for 2 min, raised to 180 °C at a rate of 5 °C/min and held for 8 min, increased to 210 °C at a rate of 2.5 °C/min, then elevated to 325 °C at a rate of 5 °C/min and was maintained for 10 min. The injector, transfer line and ion source temperatures were set to 300, 310 and 270 °C, respectively. The mass range (35–650 *m*/*z*) in full-scan mode for electron impact ionization (70 eV) was applied. The solvent delay time was set to 14 min.

### 2.4. Identification and Quantification of Metabolites

The identification of each metabolite was positively confirmed by a comparison of retention time and mass spectral data with those of a NIST/EPA/NIH Mass Spectral Library (version 2.0, National Institute of Standards and Technology, Gaithersburg, MD, USA). All the metabolites were identified by comparing mass fragments with the standard mass spectra on the commercial database NIST with a similarity of more than 70%. The calculated area of each compound was normalized by dividing the internal compound (fluoranthene) peak area to determine the semi-quantitative composition of components. Each compound was quantified against the internal standard by integrating the peak areas.

### 2.5. Determination of Proximate Composition

General component analyses were conducted using the Association of Official Analytical Chemists (AOAC) method as the standard [19]. Moisture was measured with a moisture meter (MB45, Ohaus Corporation, Zurich, Switzerland) using a 105 °C normal-pressure and high-temperature drying method. Crude protein was measured using an automatic nitrogen distillation device (Kjeltec 2200 analyzer, Foss Co. Slangerupgade, Hillerod, Denmark) following the micro-Kjeldahl nitrogen method and crude fat was measured by Soxhlet’s extraction method using an automatic crude fat extractor (Soxhlet Avanti 2050, Foss Co., Slangerupgade, Hillerod, Denmark). Furthermore, ash was quantified by a direct method at 550–600 °C using an electric furnace (Thermolyne F-48000, Bransted/Thermolyne Co., Dubuque, IA, USA). The carbohydrate content was determined by subtracting the contents of water, crude protein, crude fat and crude flour from 100 using the subtraction method.

### 2.6. Determination of Mineral Contents

After adding distilled water to 1 g of the freeze-dried powder samples according to the aging period, their volume was increased to 30 mL and they were ultrasonically extracted for 1 h; then, ion components were extracted in an oven at 85 °C for 15 h. The extract was filtered through a 0.2 µm filter and analyzed using ion chromatography (ICS-3000, Dionex, Sunnyvale, CA, USA). Among mineral nutrients, cations among inorganic nutrients were quantified using the IonPac^®^ AS20 Anion-Exchange Column, and 15 to 40 mM KOH (0–8 min: 15 mM; 8–18 min: 40 mM; 19–20 min: 15 mM) was used as the mobile phase. Anions were quantified using an IonPac^®^ CS16 Cation-Exchange Column, and 40 mM methanesulfonic acid was used as the mobile phase. The solvent flow rate was 1 mL/min, and the column temperature was fixed at 30 °C for analysis. The injection volume was 25 μL and was detected for 20 min.

### 2.7. Color Analysis

Surface color measurements of the freeze-dried powder samples according to the aging period were determined using a Hunter color reader (CR-400, Minolta Co., Osaka, Japan) on cross-sections selected from five random locations of each sample. Lightness (L value), redness (a value) and yellowness (b value) were recorded. A white standard plate with a lightness of 95.59, redness of −1.22 and yellowness of +10.07 was used as a reference.

### 2.8. pH, Reducing Sugars and Browning Intensity Analysis

For pH, reducing sugars and browning intensity analysis, 10 g of the freeze-dried powder samples according to the aging period was added to 10-fold distilled water, homogenized for 3 min at 1500 rpm with a homogenizer (PT-2100, Kinematica Ag, Littau, Switzerland) and then the supernatant was filtered. The pH was measured using a pH meter (PB 30, Sartorius, Goettingen, Germany), and the browning intensity was measured with a UV–Vis spectrophotometer (T60UV, PG Instruments, Wibtoft, UK) at an absorbance of 420 nm.

The determination of reducing sugar content was carried out using the DNS method [20]. The freeze-dried powder sample according to the aging period (1 mL) was mixed with 3 mL of the DNS reagent and shaken slightly. The mixture solution was heated in a 75 °C water bath (WBT-10, Jeongbiotech co., Incheon, Republic of Korea) for 5 min. After heating, the mixture was cooled in running water for 10 min. The absorbance at 575 nm was determined. The standard curve used glucose, and the reducing sugar content of the EG extract according to the aging period was expressed as the equivalent of D-glucose per 1 mL of the sample.

### 2.9. Extraction of EG and BEG

EG and BEG extractions were used to analyze TPC, TFC and DPPH free radical scavenging activity. After the 20-fold addition of 70% ethanol to 20 g of the freeze-dried powder samples according to the aging period, the reflux extraction method was performed and repeated 3 times for 2 h at 80 °C in a water bath. The extract was filtered and evaporated under vacuum (NVC-2100, EYELA, Tokyo, Japan) and freeze-dried (MCFD 8508, Ilshin Bio Base, Yangju, Republic of Korea) for 72 h at −40 °C.

### 2.10. Determination of Total Polyphenol Content (TPC)

TPC was determined based on the Folin–Ciocalteu method by Hillis and Swain [21]. Briefly, 150 μL of freeze-dried extract, 2400 μL of distilled water and 50 μL 2 N Folin–Ciocalteu reagent mixtures were reacted at room temperature for 3 min. Then, after 300 µL of 5% Na_2_CO_3_ was added, the mixtures were reacted in the dark for 2 h. The absorbance was measured using a UV/Vis spectrophotometer (T60UV, PG Instruments, Wibtoft, England) at 725 nm. Gallic acid was used as the standard compound, and TPC results were expressed as milligram gallic acid equivalents (GAE) per gram of extract.

### 2.11. Determination of Total Flavonoid Content (TFC)

TFC was measured based on the method developed by Davis [22], with slight modification. Briefly, 1 mL of freeze-dried extract was mixed with 90% diethylene glycol (10 mL). The mixtures were supplemented with 1 N NaOH (1 mL) and reacted at 37 °C for 1 h. The absorbance was measured at 420 nm. Rutin was used as the standard compound, and TFC results were expressed as milligram rutin equivalents (RE) per gram of extract.

### 2.12. DPPH Free Radical Scavenging Activity Assay

The DPPH radical scavenging activity assay was performed, following the method of Blois [23] with some modifications. First, 3 mL of freeze-dried extract was mixed with 1 mL of DPPH solution (1.5 × 10⁻⁴ M, in ethanol). The mixtures were incubated in darkness for 30 min at room temperature and measured at 517 nm to evaluate the absorbance using a UV/Vis spectrophotometer. The half maximal inhibitory concentration (IC_50_) indicating the concentration at which 50% of DPPH radicals were scavenged was calculated for each extract. The DPPH radical scavenging activity was calculated using the equation below:

DPPH radical scavenging activity (%) = (1 − sample absorbance/control absorbance) × 100

## 3. Statistical Analysis

All the experiments in this study were performed in triplicate, and the results are presented as the mean ± SD of three independent experiments. All the experimental results were analyzed using the SPSS program (Statistical Analysis Program, version 25, IBM Co., Armonk, NY, USA). One-way analysis of variance (ANOVA) and Duncan’s multiple range test were used at the level of significance of *p* < 0.05. The partial least square (PLS) models were created using SIMCA software (Ver 17, Umetrics, Umea, Sweden), and bivariate correlation analyses were conducted using IBM SPSS Statistics.

### 3.1. Results and Discussion

#### 3.1.1. Proximate Composition

An approximate compositional analysis was performed to identify changes in the quality-forming properties of EG during aging. The time-dependent changes in the proximate composition of the samples during aging at 70 °C and 90% relative humidity are shown in Table 1. The moisture contents of EG, BEG1, BEG2, BEG3 and BEG4 were 6.28, 15.84, 16.95, 12.23 and 13.14 g/100 g, respectively (*p <* 0.001). In this experiment, moisture absorption occurred more actively than dehumidification at the beginning of aging, and the moisture content increased until 20 days. When the aging period exceeded 20 days, moisture was lost through the heat treatment process at a high temperature of 70 °C, and the moisture absorption process decelerated, contributing to increasing the level of dehumidification. Therefore, the water content decreases after aging for 30 days because of the evaporation of free water in the food, leaving only bound water [15]. Shin et al. reported that the moisture content of garlic significantly decreased at the aging temperature of 90 °C, but the decrease in moisture content was small at 60–80 °C, indicating that the aging temperature affects the moisture content [24]. In addition, Seo reported that when garlic was aged, the moisture content increased until 10 days of aging and then decreased as the aging period elapsed, showing a similar trend to this study [5].

The crude protein content of the samples ranged from 10.61% in BEG2 to 13.24% in EG. The carbohydrate contents of EG, BEG1, BEG2, BEG3 and BEG4 were 77.90, 70.36, 69.58, 73.35 and 73.02 g/100 g, respectively (*p <* 0.001). Regarding the crude ash content, EG showed the lowest (2.59%) and BEG3 exhibited the highest (3.14%), indicating a tendency to increase with the aging period until 30 days of aging (*p <* 0.001). The crude fat content was in the range of 0.24–0.64%, and as the aging period increased, the crude fat content tended to increase (*p <* 0.001). When compared with previous studies on normal black garlic, there were differences in the proximate composition in samples. This is because there are differences in processing methods as well as ingredients between normal garlic and EG [4].

#### 3.1.2. Mineral Contents

Mineral content analysis was carried out to confirm the quality-forming characteristics of EG during the aging process. The mineral contents of the samples as a function of the aging period are shown in Table 2. Among minerals, the potassium content was the highest in all samples, and the potassium, sulfate and phosphate contents increased as the aging period elapsed (*p <* 0.001). EG had the lowest levels of potassium, sodium, magnesium, calcium, sulfate and phosphate before aging, but after aging, potassium, sodium, magnesium, calcium, sulfate and phosphate showed an increase in content. Kim et al. also reported similar results of the highest potassium and increased sodium, magnesium, calcium, sulfate and phosphate contents in BEG after aging [6]. Choi et al. measured the mineral contents of raw garlic, steamed garlic and black garlic. As a result, the mineral content of black garlic increased more than that of raw garlic, which is considered to be a result of differences in processing [7].

#### 3.1.3. Color Values and Browning Intensity

**Figure 1:**
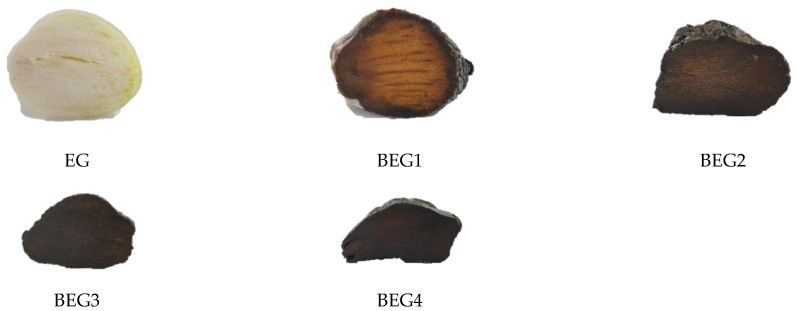
Changes in the color of EG during the aging period. Elephant garlic (EG), black elephant garlic aged for 10 days (BEG1), black elephant garlic aged for 20 days (BEG2), black elephant garlic aged for 30 days (BEG3), black elephant garlic aged for 40 days (BEG4).

Color is one of the most important psychological properties affecting consumer perceptions toward food. The browning intensity of aged garlic is affected by temperature, moisture content and reducing sugars [25]. The color values and browning intensity results of the BEG according to the aging period are shown in Table 3 and Figure 1. The L value (lightness) was 95.33 for EG, which decreased sharply to 33.25–33.61 in BEG aged for 10 days (*p <* 0.001). Consistent with the development in the dark brown appearance of aged garlic during the aging period, the a value (redness) was the lowest in EG at −2.07 and in BEG1 at 7.37, showing an initial rapid increase in redness and tending to decrease with the prolonging of the aging period (*p <* 0.001). For the b value (yellowness), EG showed the highest value at 17.16, and in BEG1, BEG2, BEG3 and BEG4, it decreased to 11.22, 4.28, 2.86 and 2.03, respectively (*p <* 0.001).

During the aging period, the browning intensity of the samples increased from 0 to 20 days and then plateaued, such that the final optical density value was 3.00 (*p <* 0.001). Polyphenol oxidase loses its activity at 50–70 °C; therefore, it is reasoned that the degree of browning increased due to nonenzymatic browning reactions between amino acids of peptides and sugars, as well as between α-amino groups of proteins and sugars [26].

#### 3.1.4. pH

The changes in the pH of the samples during the aging period are shown in Table 3. The pH of EG was 6.47, but as the aging period increased, the pH decreased significantly, and it was confirmed that it was acidic (*p* < 0.001). Najman et al. reported a similar result, that the pH of aged garlic (heated at 70 °C, 80% relative humidity, 45 days) was around 2.1–2.5 pH units lower compared with fresh garlic [27]. The pH decrease in the heated garlic sample was partially associated with the browning development upon heat treatment during the black garlic manufacturing process. The formation of carboxylic acids, which are produced by the oxidation of aldehyde groups in aldohexoses, along with the formation of acidic compounds and the decrease in basic amino acids by combining with sugars, is reported to be responsible for the decrease in pH in the browning reaction [28,29]. In addition, it has been suggested that the pH is lowered by the pyruvic acid produced by the decomposition of alliin in fresh garlic by the heat treatment process during the aging of garlic [30]. As a result of the low pH caused by the aging process, aged garlic has a comparatively long shelf life [31]. A low pH is beneficial for eliminating colonies of bacteria or fungi which cause food spoilage [32]. The decrease in pH value not only contributes to the acidic preservative action of black garlic, but also produces a sour taste and mouthfeel [33].

### 3.1.5. Reducing Sugars

At high temperatures during garlic processing, numerous nonenzymatic browning reactions occur, such as the Maillard reaction, caramelization and macromolecular degradation [8,29]. The changes in the reducing sugars of the EG during the aging period are shown in Table 3. The reducing sugars of EG, BEG1, BEG2, BEG3 and BEG4 were 0.37%, 7.16%, 9.67%, 9.23% and 9.11%, respectively (*p* < 0.001). Although the reducing sugar content initially increased with the aging period, it showed a tendency to decrease after aging for 20 days. The decrease in pH during heat treatment promotes the decomposition of sucrose into glucose or fructose, thereby increasing the content of these saccharides during the maturation of black garlic [26]. This was exemplified in the study by Choi et al., who showed that the sugar content (e.g., glucose, fructose, sucrose and maltose) increased in black garlic compared with fresh and steamed garlic [25,34]. During the initial aging of garlic at 70 °C, the rate of production of reducing sugar is faster than the rate of consumption in the Maillard reaction, which explains the increase in reducing sugar content until day 20 of aging. With further aging, reducing sugars due to consumption in the Maillard reaction decrease from day 30 [26,35].

### 3.1.6. Profiling of Metabolites in EG according to the Aging Period

In this study, to identify the metabolites of major nutrients, including amino acids, organic acids, sugars and sugar derivatives, in garlic according to the aging period, each sample was evaluated by GC/MS after derivatization. The results are shown in Table 4. In total, 41 metabolites were identified in the GC/MS datasets obtained from garlic according to the aging period. These compounds were found at various levels during the aging period and included 12 amino acids (alanine, leucine, isoleucine, valine, threonine, glycine, serine, aspartic acid, pyroglutamic acid, GABA (γ-aminobutyric acid), asparagine and glutamic acid), 10 organic acids (propanoic acid, lactic acid, glycolic acid, oxalic acid, β-lactic acid, succinic acid, glyceric acid, malic acid, 2-deoxytetronic acid and L-threonic acid), 13 sugars and sugar derivatives (D-erythro-pentofuranose, ribitol, 2-deoxy-D-erythro-pentofuranose, fructofuranose, sorbose, fructose, glucose, β-D-glucopyranose, sucrose, xylose, ribofuranose, fructopyranose and 3-α-mannobiose) and 6 others (phosphoric acid, 2-deoxypentonic acid, D-erythronic acid, 5-hydroxymethyl-2-furoic acid (5-HMFA), 2-desoxy-pentos-3-ulose and ribonic acid) (Table 4).

Among the amino acids detected, pyroglutamic acid displayed the highest content in all the samples. Serine, asparagine and glutamic acid tended to decrease as the aging period elapsed. Amino acid metabolites, such as leucine, isoleucine, threonine, glycine, aspartic acid and pyroglutamic acid, showed the lowest content in EG, increased up to 10 or 20 days and then decreased gradually with further aging. In addition, GABA, a biologically important nonprotein amino acid, showed a tendency to decrease after 20 days. According to previous studies, GABA tends to decrease due to damage and involvement of amino acids in nonenzymic browning reactions during black garlic production [36].

Among the organic acid metabolites detected, malic acid exhibited the highest content in all samples. Organic acids, such as lactic acid, glycolic acid, β-lactic acid, succinic acid, glyceric acid, 2-deoxytetronic acid and L-threonic acid, showed a tendency to increase throughout the aging period. Conversely, oxalic acid, a toxic organic acid, was the only organic acid that decreased significantly after aging and was not detected in BEG2, BEG3 or BEG4. The increased organic acid contents in BEG result from the formation of carboxylic acids, produced by the oxidation of the aldehyde groups in aldohexoses, and the decrease in basic amino acids by combining with sugars in the Maillard reaction [37,38,39].

Black garlic is rich in sugar and has a high total sugar content of 60–70% per mass [40]. During the aging process, garlic polysaccharides are broken down into monosaccharides, including glucose and fructose [41]. Fructose recorded the highest content of all the sugar and sugar derivative metabolites detected. Sugars 2-deoxy-D-erythro-pentofuranose and xylose tended to increase throughout aging. Most of the sugar and sugar derivative metabolites (D-erythro-pentofuranose, ribitol, fructopyranose, fructose, sorbose, glucose, β-D-glucopyranose, ribofuranose, fructofuranose and 3-α-mannobiose) increased up to 20 days of aging, and then decreased subsequently. Among the sugar metabolites, sucrose, a disaccharide, had the highest content in EG and was the only sugar metabolite to decrease as aging progressed.

As mentioned above, six metabolites classified as others were also detected. These six metabolites showed a tendency to increase with the aging period, and 2-deoxypentonic acid, D-erythronic acid, 5-HMFA, 2-desoxy-pentos-3-ulose and ribonic acid were not detected in EG.

In a study on the antioxidant activity of the Maillard reaction products, most amino acids, except cysteine and tryptophan, and sugars, such as fructose and glucose, produce melanoidins, dark browning polymeric compounds, due to the Maillard reaction, which improve antioxidant activity [42]. In the present metabolite experiment, most of the sugars and amino acids required for the Maillard reaction increased up to 20 days of aging and decreased at 30 days.

Differences in metabolites appearing after the aging of foods are related to changes in the sensory properties of foods, such as color or flavor, as well as differences in antioxidant effects [41]. The sugar and amino acid contents of aged BEG were relatively high; thus, it is expected that the sweet and sour taste will be enhanced.

### 3.1.7. TPC, TFC and DPPH Free Radical Scavenging Activity

Garlic is one of the richest sources of phenolic compounds among the common vegetables in the human diet [43]. To determine the antioxidant properties of black garlic during aging, we focused on the analysis of TPC and TFC (Table 5). TPC (11.84 to 27.08 mg GAE/g) and TFC of BEG (2.48 to 8.75 mg RE/g) were not only significantly higher than those of EG (4.61 mg GAE/g and 0.86 mg RE/g), but also increased significantly until day 30 of aging, before decreasing after that (*p* < 0.001). According to Shin et al., TPC and TFC were significantly higher in black garlic than in raw garlic [24]. In addition, TPC increased significantly until 21 days of aging and TFC until 28 days of aging, before decreasing after that, showing a similar trend to that of this study [26]. When compared with EG, BEG has higher antioxidant activity due to conversion of antioxidant compounds such as bioactive alkaloids and flavonoid compounds during the aging process [15]. BEG also contains bioactive compounds, such as phenols, flavonoids, pyruvate, thiosulfate, S-allylcysteine and S-allylmercaptocysteine. The heating process of EG leads to the Maillard reaction, creating the typical dark brown color, and produces antioxidant compounds [10,15,24,25]. According to Xu et al., the heat treatment of the phenolic compounds increases the fraction of free phenolic acids but decreases the ester, glycoside and ester-bound fractions, leading to an increase in free phenol forms [43]. In addition, another probable reason for the increase in the phenolic content in the heated sample is the decrease in/inhibition of enzymatic oxidation involving the antioxidant compounds in the raw plant material [44]. Kang et al. reported that the TPC in BG can be affected by several factors, such as temperature, pressure, time, humidity, etc., and duration of aging [45].

The results of DPPH free radical scavenging activity are expressed as IC_50_, and the lower IC_50_ value indicates stronger antioxidant activity. The IC_50_ values of DPPH free radical scavenging activity were 5927.86 μg/mL for EG, 992.13 μg/mL for BEG1, 591.36 μg/mL for BEG2, 465.46 μg/mL for BEG3 and 524.01 μg/mL for BEG4, as shown in Table 6 (*p* < 0.001). The DPPH radical scavenging activity ranged from 20.27% in EG to 90.98% in BEG3 at 1 mg/mL, indicating a tendency to increase with the aging period until 30 days, as shown in Table 6 (*p* < 0.001). In a previous study of black garlic, the DPPH activity increased intensively (about twofold) until day 21 of aging, then decreased slightly after that, showing a similar trend to the present study [7]. When garlic is aged, the color changes to black due to the Maillard reaction, which reacts with sugar by heat, and amino acids are denatured, resulting in antioxidant effects. The 5-hydroxy methyl furfural, lactic acid, adenosine and uridine products produced after the Maillard reaction increase during processing and give an antioxidant effect [46]. The increase in DPPH free radical scavenging activity after aging is attributed to various bioactive substances such as phenols and flavonoids present in elephant black garlic [25]. Soobrattee et al. reported that the antioxidant activity increased when the TPC increased. BEG3 showed the highest results in TPC, TFC and DPPH free radical scavenging activity [47]. Based on the above results of antioxidant compounds and antioxidant activities, we propose that the optimum aging period for maximizing the antioxidant properties of BEG is 30 days.

### 3.1.8. Correlation between Metabolite Variation and Antioxidant Activities of EG According to Aging Period

**Figure 2:**
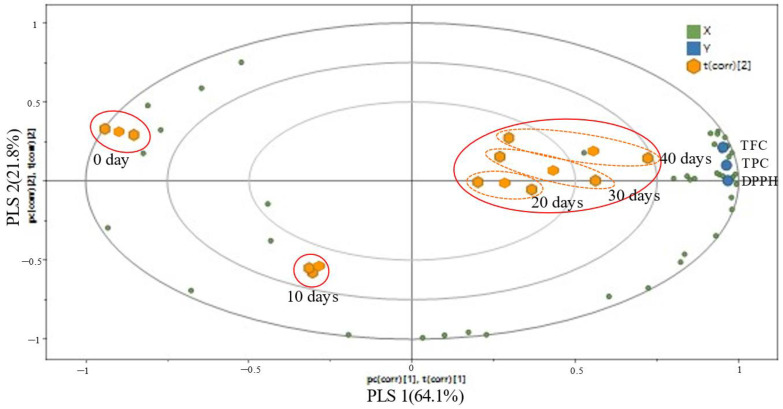
Partial least square (PLS) biplot between metabolites (*X*) and antioxidant activities (*Y*) of EG according to the aging period.

PLS was performed to determine the correlation between the metabolic variation and antioxidant activity of EG according to the aging period. In the PLS biplot for EG aged for different times, PLS components 1 and 2 together accounted for 85.9% of the total variance: 64.1% and 21.8%, respectively. The parameter of the cross-validation modeling was PLS component 3, with *R*^2^*X* = 0.935, *R*^2^*Y* = 0.961, and *Q*^2^ (cumulative) = 0.879. In this study, a single graphical representation, which combined the score and loading plots, was created, as shown in the PLS biplot. The PLS biplot shows the pair-wise correlation between all variables (*X* and *Y*), illustrating the association between antioxidant activities and metabolites. In addition, the chemical shift of the metabolites was represented by the *X* variables, and antioxidant activities were represented by the *Y* variables (Figure 2). In Figure 2, the PLS biplot shows a clear separation into three groups, strongly correlated with antioxidant activity. EG samples aged for 20, 30 and 40 days are located in the upper right of the PLS biplot, and antioxidant activity variables (TPC, TFC and DPPH free radical scavenging activity) were found near these samples. This indicates that EG samples aged for 20, 30 and 40 days had higher antioxidant activity than samples aged for 0 and 10 days. These results suggest that some new antioxidant components might have been produced during the aging of EG.

When assessing a common metabolite with a correlation matrix of 0.8 or higher according to PLS, organic acids (lactic acid, glycolic acid, β-lactic acid, succinic acid, glyceric acid and L-threonic acid), sugars and sugar derivative metabolites (2-deoxy-D-erythro-pentofuranose, glucose, xylose, ribofuranose and fructofuranose) and other metabolites (2-deoxypentonic acid, D-erythronic acid, 2-desoxy-pentos-3-ulose, 5-HMFA and ribonic acid) had a substantial influence on antioxidant activity.

In particular, lactic acid is the major organic acid in BEG, as found by metabolite analysis; therefore, lactic acid may be responsible for the unique flavor of black garlic [48]. Furthermore, lactic acid is also a strong antioxidant, which could have contributed to the strong antioxidant capacity of black garlic [49].

In metabolite analysis, 5-HMFA, which is the main metabolite of 5-hydroxymethylfurfural (5-HMF), was not found in EG, whereas the amount of 5-HMFA increased during the processing of EG into BEG, reaching 10-fold the 5-HMF content at 40 days of the aging process compared with aging for 10 days (Table 4). 5-HMF is a five-carbon-ring aromatic aldehyde which does not naturally occur in fresh foods but can be formed in sugar-containing foods during thermal treatments [50]. Its formation derives from the catalytic dehydration of Amadori products during the Maillard reaction at high temperatures and the direct degradation of hexose sugars in an acidic environment [39]. 5-HMF exhibits a range of biological and pharmacological activities, such as antioxidant [50], antitumor [51] and anti-inflammatory properties [52]. However, 5-HMF may be cytotoxic at high concentrations and causes irritation to tissues and internal organs of the human body, with mutagenicity and carcinogenicity in vivo [53,54], although the results of epidemiological investigations have not yet confirmed the potential association of 5-HMF with cancer risks in humans [55]. 5-HMF not only affects the biological activity (one major antioxidant in black garlic) but also the sensory characteristics of black garlic (with bitterness at a relatively higher concentration) [53]. The formation of 5-HMF in foods is highly dependent on processing and storage conditions, such as temperature and pH [56]. As a crucial intermediate in the Maillard reaction with a close correlation with the browning rate of food, 5-HMF can be an important factor for black garlic in setting the aging period [53].

## 4. Conclusions

This study measured the content of quality indicators according to the aging period during the processing of EG. The aging period significantly affected the quality of BEG, including the antioxidant activity and content of various substances in garlic. Browning was shown to proceed with aging due to browning reactions, resulting in high antioxidant activity. In addition, the accumulation of reducing sugars is relatively high in aged BEG and the sweetness and umami taste are strengthened due to the increases in the sugars and amino acids. In addition, as the organic acids increased, the pH decreased, and improvements in storage properties were expected. In the aging process of EG, compounds with strong antioxidant properties were formed due to nonenzymatic browning reactions, and the content of antioxidant components, including TPC and TFC, increased significantly up to 30 days. Thus, it was confirmed that the optimal ripening period for maximizing the antioxidant properties was 30 days. The PLS results confirmed the relationship between the antioxidant activity and metabolites of EG according to the aging period, showing that some metabolites, such as lactic acid, glycolic acid, 2-deoxy-D-erythro-pentofuranose, ribofuranose, 2-deoxypentonic acid and 5-HMFA, were highly correlated with strong antioxidant activity. As such, we expect that the difference in metabolites after aging was related to changes in the sensory characteristics of garlic, such as color and taste, and affected the antioxidant activity. These results can contribute to understanding the role of the appropriate processing period in shaping the quality of BEG. However, achieving quality in BEG production is a considerably complex process which is influenced by several factors in addition to the aging period, such as temperature and relative humidity, during the aging process. Further efforts to elucidate the effects of these factors on the quality of BEG products are required in future studies.

## Figures and Tables

**Table 1:** Proximate composition of EG powders according to the aging period.

Treatments ^(1)^	Proximate Composition (g/100 g, DW)				
	Moisture	Crude Protein	Crude Fat	Crude Ash	Carbohydrate
EG	6.28 ± 0.01 ^e^	13.24 ± 0.04 ^a^	0.24 ± 0.02 ^c^	2.59 ± 0.03 ^d^	77.90 ± 0.02 ^a^
BEG1	15.84 ± 0.01 ^b^	10.96 ± 0.02 ^c^	0.04 ± 0.01 ^e^	2.85 ± 0.01 ^c^	70.36 ± 0.00 ^d^
BEG2	16.95 ± 0.03 ^a^	10.61 ± 0.08 ^e^	0.10 ± 0.00 ^d^	2.87 ± 0.01 ^c^	69.58 ± 0.06 ^e^
BEG3	12.23 ± 0.03 ^d^	11.29 ± 0.04 ^b^	0.30 ± 0.03 ^b^	3.14 ± 0.02 ^a^	73.35 ± 0.04 ^b^
BEG4	13.14 ± 0.06 ^c^	10.81 ± 0.05 ^d^	0.64 ± 0.04 ^a^	3.04 ± 0.01 ^b^	73.02 ± 0.00 ^c^

All values are the mean ± SD (*n* = 3). ^(1)^ Elephant garlic (EG), black elephant garlic aged for 10 days (BEG1), black elephant garlic aged for 20 days (BEG2), black elephant garlic aged for 30 days (BEG3), black elephant garlic aged for 40 days (BEG4). ^a–e^ Values with different letters within a column differ significantly by Duncan’s multiple range test (*p* < 0.05).

**Table 2:** Mineral contents of EG powders according to the aging period.

Treatments ^(1)^	Mineral Contents (mg/100 g DW)							
	Sodium	Potassium	Magnesium	Calcium	Chlorine	Sulfate	Ammonium	Phosphate
EG	1.93 ± 0.09 ^c^	981.59 ± 0.37 ^e^	31.74 ± 1.55 ^e^	0.90 ± 0.20 ^e^	78.49 ± 0.38 ^b^	11.88 ± 0.33 ^d^	149.01 ± 0.02 ^b^	133.81 ± 1.39 ^e^
BEG1	1.78 ± 0.06 ^c^	1184.41 ± 22.30 ^d^	82.51 ± 0.77 ^a^	8.12 ± 0.44 ^d^	58.54 ± 1.65 ^d^	30.84 ± 0.16 ^c^	161.81 ± 1.23 ^a^	672.73 ± 4.35 ^d^
BEG2	1.71 ± 0.05 ^c^	1238.25 ± 1.96 ^c^	65.61 ± 2.23 ^d^	10.58 ± 0.09 ^c^	62.95 ± 2.65 ^c^	31.07 ± 1.06 ^c^	118.66 ± 1.37 ^c^	807.35 ± 3.28 ^c^
BEG3	9.82 ± 0.18 ^a^	1290.54 ± 14.61 ^b^	76.19 ± 1.14 ^c^	21.17 ± 0.42 ^a^	79.54 ± 1.43 ^b^	44.15 ± 1.29 ^b^	96.67 ± 1.62 ^d^	934.04 ± 2.16 ^b^
BEG4	2.29 ± 0.23 ^b^	1334.28 ± 8.16 ^a^	80.84 ± 6.09 ^ab^	17.80 ± 0.33 ^b^	84.45 ± 1.47 ^a^	57.73 ± 0.31 ^a^	70.04 ± 0.56 ^e^	1034.63 ± 2.76 ^a^

All values are the mean ± SD (*n* = 3). ^(1)^ Elephant garlic (EG), black elephant garlic aged for 10 days (BEG1), black elephant garlic aged for 20 days (BEG2), black elephant garlic aged for 30 days (BEG3), black elephant garlic aged for 40 days (BEG4). ^a–e^ Values with different letters within a column differ significantly by Duncan’s multiple range test (*p* < 0.05).

**Table 3:** Color values, browning intensity, pH and reducing sugar contents of EG according to the aging period.

Treatments ^(1)^	L Value	a Value	b Value	Browning Intensity (420 nm OD)	pH	Reducing Sugar Content (%)
EG	95.33 ± 0.19 ^a^	−2.07 ± 0.06 ^e^	17.16 ± 0.11 ^a^	0.40 ± 0.01 ^d^	6.47 ± 0.03 ^a^	0.37 ± 0.01 ^d^
BEG1	33.25 ± 0.81 ^b^	7.37 ± 0.08 ^a^	11.22 ± 0.52 ^b^	1.19 ± 0.01 ^c^	4.57 ± 0.01 ^b^	7.16 ± 0.13 ^c^
BEG2	33.36 ± 0.64 ^b^	6.10 ± 0.17 ^b^	4.28 ± 0.74 ^c^	2.83 ± 0.01 ^b^	4.04 ± 0.02 ^c^	9.67 ± 0.30 ^a^
BEG3	33.41 ± 0.45 ^b^	3.66 ± 0.06 ^c^	2.86 ± 0.41 ^d^	3.00 ± 0.00 ^a^	3.81 ± 0.01 ^d^	9.23 ± 0.18 ^b^
BEG4	33.61 ± 0.50 ^b^	2.35 ± 0.17 ^d^	2.03 ± 0.31 ^d^	3.00 ± 0.00 ^a^	3.68 ± 0.01 ^e^	9.11 ± 0.07 ^b^

All values are mean ± SD (*n* = 3). ^(1)^ Elephant garlic (EG), black elephant garlic aged for 10 days (BEG1), black elephant garlic aged for 20 days (BEG2), black elephant garlic aged for 30 days (BEG3), black elephant garlic aged for 40 days (BEG4). ^a–e^ Values with different letters within a column differ significantly by Duncan’s multiple range test (*p* < 0.05).

**Table 4:** Tentatively identified compounds of EG by GC/MS according to the aging period.

Compounds	RT ^(1)^ (min)	Treatment ^(2)^					TMS ^(3)^	Quantitative Ion
		EG	BEG1	BEG2	BEG3	BEG4		
Amino acids								
Alanine	17.79	521.19	70.45	322.18	199.19	227.72	2	116
Leucine	19.53	24.50	601.78	316.90	185.07	91.65	1	86
Isoleucine	20.18	ND ^(4)^	316.97	180.58	141.06	84.94	1	86
Valine	21.17	547.26	132.37	352.08	194.34	179.96	2	144
Threonine	23.44	20.88	134.10	62.73	27.29	10.51	2	117
Glycine	23.78	247.32	209.74	392.89	327.31	258.53	3	174
Serine	25.17	99.29	28.99	57.87	16.66	9.79	3	204
Aspartic acid	26.97	27.51	213.12	194.96	199.18	120.05	2	160
Pyroglutamic acid	29.55	2670.61	25,623.1	34,005.24	28,167.81	24,740.50	2	156
*γ*-Aminobutyric acid	29.74	411.97	317.87	511.51	205.84	103.26	3	174
Asparagine	31.84	811.77	713.83	209.12	82.64	ND	2	75
Glutamic acid	32.74	153.38	78.52	78.83	42.56	37.46	3	246
Organic acids								
Propanoic acid	16.15	49.65	532.77	328.54	187.20	143.59	2	147
Lactic acid	16.45	34.34	268.72	919.14	1236.65	1380.65	2	147
Glycolic acid	16.97	21.55	2020.76	3768.23	4811.96	5397.26	2	147
Oxalic acid	18.96	930.82	14.74	ND	ND	ND	2	147
*β*-Lactic acid	19.07	ND	315.19	1537.15	2640.01	3418.81	2	147
Succinic acid	24.00	34.68	230.71	307.44	438.10	547.67	2	147
Glyceric acid	24.35	12.87	624.24	1476.12	2246.77	2836.04	3	147
2-Deoxytetronic acid	27.05	ND	21.36	51.36	69.65	84.17	3	233
Malic acid	28.49	1928.60	5669.57	4980.11	5093.08	4800.89	3	147
L-Threonic acid	29.91	21.50	1144.63	1250.34	1419.17	1454.97	4	147
Sugars and sugar derivatives								
D-Erythro-pentofuranose	33.95	35.35	36.28	39.95	27.34	17.79	3	245
Ribitol	35.82	19.92	197.26	135.73	83.81	59.37	5	103
2-Deoxy-D-erythro-pentofuranose	39.02	23.35	43.66	181.76	237.03	300.97	3	129
Fructopyranose	40.93	ND	12,546.04	79,821.30	46,575.95	40,806.23	5	204
Fructose	43.50	1141.52	781,178.8	824,092.8	713,242.3	741,031.80	5	103
Sorbose	44.27	ND	1522.51	6331.34	5254.07	5251.97	5	103
Glucose	44.47	132.31	16,917.33	80,072.42	52,372.73	48,026.24	5	204
*β*-D-Glucopyranose	48.28	122.19	23,690.27	109,373.60	72,818.34	68,102.54	5	204
Sucrose	63.98	65,372.61	138,504.1	4686.93	113.43	ND	8	361
Xylose	64.31	ND	66.38	575.00	718.03	742.80	5	103
Ribofuranose	64.40	1109.61	28,238.09	55,670.27	49,486.07	53,381.66	4	217
Fructofuranose	64.72	79.16	45,745.33	277,550	187,077.3	157,197.2	5	217
3-*α*-Mannobiose	65.69	27.64	233.66	729.25	585.78	597.90	8	103
Others								
Phosphoric acid	22.76	396.49	19,709.66	33,735.43	39,475.58	45,228.11	3	299
2-Deoxypentonic acid	26.40	ND	37.91	103.05	96.86	99.32	2	103
D-Erythronic acid	27.09	ND	41.86	104.95	177.45	229.67	2	103
5-Hydroxymethyl-2-furoic acid	30.33	ND	10	114.01	186.82	211.84	2	271
2-Desoxy-pentos-3-ulose	37.99	ND	160.35	508.06	576.26	695.77	2	231
Ribonic acid	39.16	ND	170.71	253.27	265.49	281.77	5	292

^(1)^ RT: retention time. ^(2)^ Elephant garlic (EG), black elephant garlic aged for 10 days (BEG1), black elephant garlic aged for 20 days (BEG2), black elephant garlic aged for 30 days (BEG3), black elephant garlic aged for 40 days (BEG4). ^(3)^ TMS: trimethylsilylation. ^(4)^ ND: not detected. All values are concentration; fluoranthene equivalent μg/g, mean values (*n* = 3).

**Table 5:** Antioxidants of EG according to the aging period.

Treatments ^(1)^	TPC (mg GAE ^(2)^/g)	TFC (mg RE/g)	DPPH Free Radical Scavenging Activity (%)
EG	4.62 ± 0.48 ^b^	0.86 ± 0.03 ^e^	20.27 ± 0.13 ^e^
BEG1	11.84 ± 0.14 ^b^	2.48 ± 0.05 ^d^	50.49 ± 0.47 ^d^
BEG2	23.43 ± 0.41 ^a^	7.27 ± 0.10 ^c^	82.49 ± 0.26 ^c^
BEG3	27.08 ± 0.14 ^a^	8.75 ± 0.21 ^a^	90.98 ± 0.23 ^a^
BEG4	25.10 ± 0.71 ^a^	8.39 ± 0.25 ^b^	85.63 ± 0.24 ^b^

All values are the mean ± SD. ^(1)^ Elephant garlic (EG), black elephant garlic aged for 10 days (BEG1), black elephant garlic aged for 20 days (BEG2), black elephant garlic aged for 30 days (BEG3), black elephant garlic aged for 40 days (BEG4). ^(2)^ GAE: gallic acid equivalents, RE: rutin equivalents. ^a–e^ Values with different letters within a column differ significantly by Duncan’s multiple range test (*p* < 0.05).

**Table 6:** DPPH free radical scavenging activities of EG according to the aging period.

Treatments ^(1)^	DPPH Free Radical Scavenging Activity IC_50_^(2)^
	(μg/mL)
EG	5927.86 ± 113.72 ^a^
BEG1	992.13 ± 7.29 ^b^
BEG2	591.36 ± 7.71 ^c^
BEG3	465.46 ± 5.27 ^d^
BEG4	524.01 ± 8.10 ^cd^
Trolox	3.03 ± 0.10 ^e^
Ascorbic acid	2.57 ± 0.03 ^e^

All values are the mean ± SD. ^(1)^ Elephant garlic (EG), black elephant garlic aged for 10 days (BEG1), black elephant garlic aged for 20 days (BEG2), black elephant garlic aged for 30 days (BEG3), black elephant garlic aged for 40 days (BEG4). ^(2)^ The IC_50_ values were calculated by linear regression analysis. ^a–e^ Values with different letters within a column differ significantly by Duncan’s multiple range test (*p* < 0.05).

## Data Availability

The data from the current study are available from the corresponding author and first author of the article on reasonable request.

## Abbreviations

5-HMF	5-Hydroxymethylfurfural
5-HMFA	5-Hydroxymethyl-2-furoic acid
BEG	Black elephant garlic
BEG1	Elephant garlic aged for 10 days
BEG2	Elephant garlic aged for 20 days
BEG3	Elephant garlic aged for 30 days
BEG4	Elephant garlic aged for 40 days
BSTFA	*N, O*-Bis trimethylsilyl trifluoroacetamide
DNS	3,5-Dinitrosalicylic acid
DPPH	2,2-Diphenyl-1-picrylhydrazyl
EG	Elephant garlic
GABA	γ-Aminobutyric acid
GAE	Gallic acid equivalents
GC/MS	Gas chromatography mass spectrometry
NIST	National Institute of Standards and Technology
PCA	Principal component analysis
PLS-DA	Partial least square discrimination analysis
RE	Rutin equivalents
TFC	Total flavonoid content
TPC	Total polyphenol content
